# Ali-Bey - an open collaborative georeferencing web application

**DOI:** 10.3897/BDJ.10.e81282

**Published:** 2022-04-28

**Authors:** Arnald Marcer, Agustí Escobar, Víctor Garcia-Font, Francesc Uribe

**Affiliations:** 1 CREAF, E08193 Bellaterra (Cerdanyola del Vallès), Catalonia, Spain CREAF E08193 Bellaterra (Cerdanyola del Vallès), Catalonia Spain; 2 Universitat Autònoma de Barcelona, E 08193, Bellaterra (Cerdanyola del Vallès), Catalonia, Spain Universitat Autònoma de Barcelona E 08193, Bellaterra (Cerdanyola del Vallès), Catalonia Spain; 3 Universitat Oberta de Catalunya (UOC), Rambla del Poblenou 156. 08018 Barcelona, Spain Universitat Oberta de Catalunya (UOC) Rambla del Poblenou 156. 08018 Barcelona Spain; 4 CYBERCAT-Center for Cybersecurity Research of Catalonia, Rambla del Poblenou 156. 08018 Barcelona, Spain CYBERCAT-Center for Cybersecurity Research of Catalonia Rambla del Poblenou 156. 08018 Barcelona Spain; 5 Museu de Ciències Naturals de Barcelona, Barcelona, Spain Museu de Ciències Naturals de Barcelona Barcelona Spain

**Keywords:** georeferencing, web application, site name versioning, collaborative database, digital specimens, natural history collections, traceability, Museu de Ciències Naturals de Barcelona

## Abstract

**Background:**

Georeferencing preserved specimens represents a major effort at the Museu de Ciències Naturals de Barcelona (MCNB), given the available resources and limited staff that can be allocated to the task. Georeferencing is a labour-intensive and hard-to-automate task that requires software tools that can help in making it as efficient as possible. The tool we present, Ali-Bey, has been slowly developed over 15 years and its functionalities have been gradually built in a process of development, testing, use in production and refinement, rather than as a single development cycle out of a comprehensive specifications requirement document. At the start, the MCNB could not find a tool that fully satisfied the requirements listed as essential and made the decision to develop a custom tool. At the end, the initiative has proved successful since it has delivered a new georeferencing tool that meets the MCNB's needs, all in a context of yearly scarce availability of funds. The tool has been gradually matured and developed over the years, in line with the scarce financing. Only recently, after reaching a notable set of novel features, we considered to release it as an open-source project. The MCNB has supported its development up until this date and decided to open it in order to give the NHC community the opportunity to contribute to its development.

**New information:**

We present the software tool Ali-Bey that provides new functionality for the georeferencing of specimens in Natural History Collections, namely the possibility of cooperation between different institutions, the traceability of georeferences and the capability of managing different versions of a same site name, namely for historical reasons. The tool is an open-source web application implemented in Python and the Django framework that leverages other commonly-used specialised geodatabase and map server tools. An API provides access to the geodatabase to externally-developed tools. In addition, for an easy installation, the tool is provided as a multi-container Docker application.

## Introduction

Institutions holding Natural History Collections (NHC) have recently embarked on new and demanding digitisation projects that can provide important societal benefits in research, conservation, education and outreach (Nelson and Ellis 2018). Usually, these projects represent additional work on top of the everyday curation, research and dissemination tasks carried out at these institutions, without receiving sufficient additional funding. Moreover, this is greatly aggravated by the sheer number of specimens held at these institutions worldwide, with estimates in the order of a few billion specimens (Ariño 2010,Marcer et al. 2021b). Large or small, each of these institutions is in charge of a number of specimens that usually far exceeds the maintenance capacity of their respective staff. Small institutions with limited staff may be in custody of tens of thousands of specimens, while large institutions with a greater number of staff may also be overwhelmed by their collection sizes going up to millions, tens of millions or even hundreds of millions of specimens. For instance, only 6% of the millions of specimens at the Natural History Museum London are available online (Weston 2021).

Assigning coordinates to preserved specimens of NHC is an important task that allows us to relate these specimens to the environmental conditions in which they were collected, presumably their living habitat or ecological niche. Once set in their ecological context, these specimens can help address fundamental questions in ecology, evolution and societal pressing issues, such as global change (Hedrick et al. 2020). This is accomplished with a process called retrospective georeferencing (Murphey et al. 2004, Chapman and Wieczorek 2020), which is an important part of the digitisation process. It involves the interpretation of text-locality descriptions into pairs of coordinates plus metadata information.

Retrospective georeferencing is a skill-demanding, labour-intensive and hard-to-automate task (Guralnick et al. 2006, Chapman and Wieczorek 2020) and it requires the dedication of considerable resources, especially in terms of staff time. Moreover, multiplication of efforts occur when different institutions georeference the same site name unbeknown to each other, since they may have specimens collected at the same locality. According to a recent survey (Marcer et al. 2021a), georeferencing can represent a significant fraction of the effort dedicated to the digitisation of collections. The only exceptions are specimens collected with the help of GPS technology, for which the GPS device can directly provide their georeferencing data. However, these represent only a tiny fraction of the world’s preserved specimens, coming from recent collection projects (Marcer et al. 2021a). For example, Blagoderov et al. 2017 estimated an average cost of about 0.105 British pounds (0.17 Euros in 2017) to georeference a single specimen; i.e. about 170 000 Euros to georeference 1 million specimens.

The job of georeferencing entails the need to enter, store, view and query alphanumeric and spatial data, navigate and query reference digital cartography, perform spatial operations, manage user access and permissions, work collaboratively and make results public and easily accessible. Software applications can provide tools that can greatly help georeferencers and their institutions in this undertaking.

The *Museu de Ciències Naturals de Barcelona (MCNB)* holds a collection of about three million preserved samples of specimens (MCNB, Museu de Ciències Naturals de Barcelona 2020). Georeferencing constitutes a major effort for the institution, given the limited staff and time that can be allocated to the task. The Museum's collection management software did not have the above-listed required functionality to effectively georeference the collection. Other existing collection management software (e.g. Arctos, Specify, Symbiota etc.) also did not meet all the requirements that were listed as essential. This led to the decision to develop custom software that could fill the needs. To this date, the MCNB has managed to georeference just over 5% of their records, with just under 2,000,000 records still in the queue.

Usually, georeferencing procedures are supported by external and specific tools, like GEOLocate (Rios and Bart 2010). Nevertheless, MCNB looked for more functionalities: easy reuse of previously-obtained data, traceability of estimations and the option of managing versions for the same locality. This paper describes *Ali-Bey*, the georeferencing open-source software platform of MCNB, named after the Arab name of Domènec Badia, a Catalan explorer of northern Africa in the late 18th and early 19th centuries (https://en.wikipedia.org/wiki/Ali_Bey_el_Abbassi). *Ali-Bey* is the tool currently used at MCNB for all matters regarding the georeferencing of NHC.

## Project description

### Title

Ali Bey - an open collaborative georeferencing web application.

### Design description

Ali-Bey is a web application aimed at providing support in the management of the georeferencing process, i.e. the assignment of coordinates to textual descriptions of site names, where preserved specimens from natural history collections were collected. It provides tools to help in determining georeferences, assigning metadata and querying, visualising, storing and publishing them. Site names can be hierarchically organised in a tree-like structure and they can be versioned, i.e. newer improved versions of georeferenced site names are kept on top of all their older versions. The application also provides user administration capabilities and the possibility of collaborating between institutions in order to sum up resources towards a common better list of georeferenced site names. Georeferenced sites can be made publicly available through a public API. The following list describes its main features.

**Latest version** 1.0.0

**Features**

Hierarchical structure

**Figure 1.**
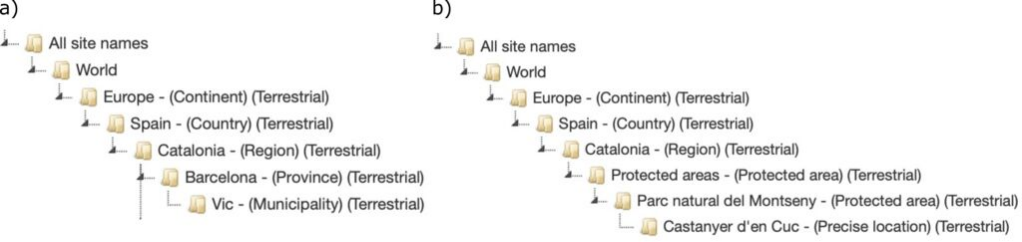
Example of two site names hierarchies administrative: a) hierarchy based on administrative units and, b) based on administrative units and protected areas.

All site names descend from a top node called *World.* Each site name can be assigned to a parent node and each node can be georeferenced, i.e. each node can be in itself a site name. For instance, the municipality *Vic* is a site name nested under the county node *Osona*, the region node *Catalonia*, the country node *Spain*, the continent node *Europe* and the top node *World*. All of these nodes can also have their corresponding set of coordinates and metadata. The hierarchy can be based on political-administrative entities as the above-mentioned example or on other user-defined hierarchies, such as physical or ecological features. This hierarchical structure not only allows the easy retrieval of all georeferenced site names for a specific world region, but also the assignment of editing permissions for different parts of the world to different users. The site names hierarchy is shown within the application in a tree-like control with which users can interact (Fig. 1).

Site names with a terrestrial/aquatic border may deserve two distinguishable georeferencing estimations: one for the terrestrial surface and another for georeferencing collecting places for aquatic organisms. For instance, fishes caught off the coast of Barcelona.

Versioned locations

**Figure 2.**
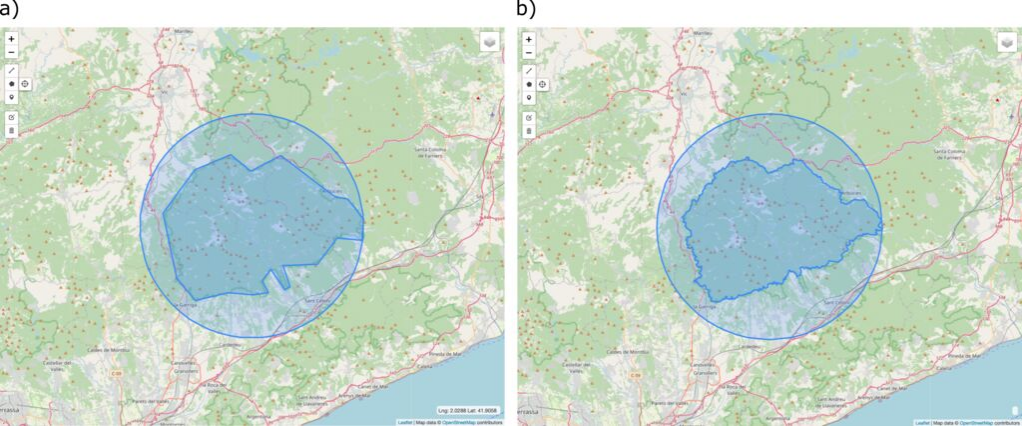
The Montseny protected area georeferenced with a polygon with its uncertainty circle: a) an example of a first coarse polygon version of the protected area; b) a second improved version of the polygon representing the georeference, also with its uncertainty circle.

Georeferenced site names are versioned, i.e. historical extents or newer improved versions of a given georeferenced site name are kept on top of the older ones (Fig. 2). Improvements may come because better interpretations of the same textual description of a locality or because better cartography, which allows for better georeferencing, have become available. Thus, versioned georeferenced site names refer to sets of different interpretations of a given location, chronologically ordered and, eventually, improved towards the more recent record. Since all georeferenced versions of a given site name are kept in the database, no past derivative work using old versions of a georeference will lose its links to the set of coordinates and uncertainty used at the time it was done. The history of each georeference is kept within the system and a simple look-up table with a customised vocabulary allows the management of the version deployment.

Coordinates and uncertainty

Uncertainty is a measure of how close an estimated measure is to its real value (Chapman and Wieczorek 2020). In the case of georeferencing, the uncertainty represents a boundary of error around an estimated coordinate for a given location or site name; i.e. the true coordinate of the site name lies somewhere within the boundary error. In the case of site names represented by graphical objects other than points, i.e. lines or polygons, the uncertainty is defined following the point-radius method (Wieczorek et al. 2004;Chapman and Wieczorek 2020).

The coordinates and uncertainty representing a given location can be entered in three alternative ways: a) they can be determined externally and manually entered into the system through a set of textbox controls, b) a spatial object representing the location can be directly entered using the built-in map viewer which allows for digitising points, lines and polygons and c) by importing a shapefile. Both in b) and c) cases, *Ali-Bey* will automatically calculate the centroid and the spatial uncertainty derived from the spatial object representing the site name.

Georeferencing resources

Resources used in the georeferencing process (e.g. maps, gazetteers, web map servers etc.) can be entered and stored in the system’s database. They can be assigned a spatial object representing the geographical extent covered by the resource. Hence, georeferencers can find which resources are available for the part of the world where a given specimen was collected. Additionally, each georeferenced site name version that is entered can be tagged with the resource used in the georeferencing process. Thus, any site name georeference history can be traced back to the georeferencing resources used in the process. In the case of web map servers, they can be registered with their corresponding URL or they can be created by importing a spatial file, such as a TIFF or shapefile. In the latter case, the spatial file is automatically entered into the system’s map server and made available as an additional layer in the map viewer.

Map viewer

**Figure 3.**
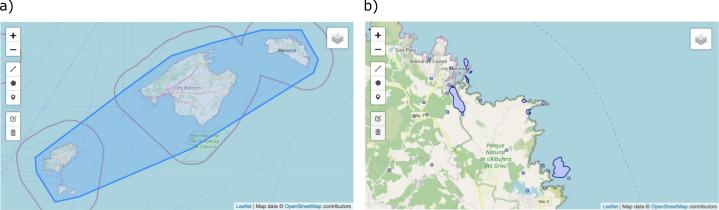
Detail of the map viewer for: a) georeferencing resources and b) site names. The polygon encompassing the georeferencing resource “Nomenclator of Site Names of the Balearic Islands” is shown in a) and a detailed view of the georeferenced site names of the eastern part of the Island of Minorca is shown in b). Tools for zooming and editing can be seen on the left side of the viewer and for layer selection on the top-right corner.

The application provides navigation map windows for visualising, querying and editing both geographical resources and site names (Fig. 3). They can be displayed on top of free online reference cartography, such as Microsoft Bing Maps and OpenStreetMaps. In the case of georeferencing resources, the limits of their geographical extent are shown as a multipolygon object. For georeferenced site names, their spatial representation objects (e.g. a municipality boundary or a river segment), their centroids and their corresponding uncertainty boundaries are shown.

Querying and filtering

**Figure 4.**
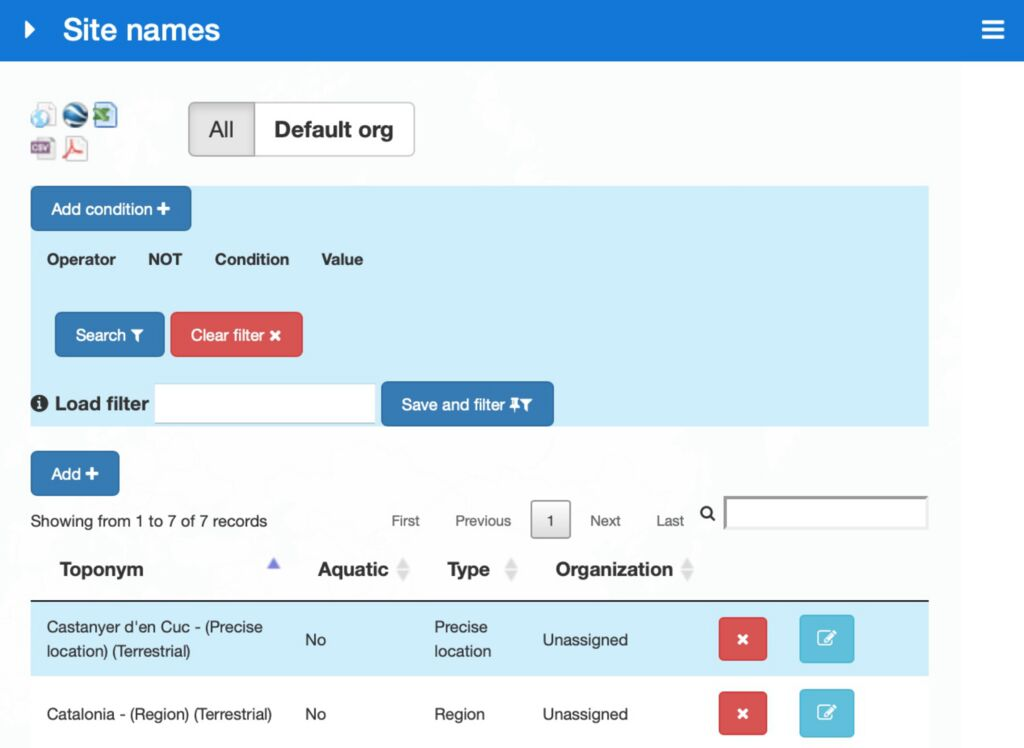
Tool for filtering and exporting site names.

Site names and georeferencing resources can be queried by clicking on them in their corresponding map viewer windows and their associated information can be accessed by clicking on the corresponding icon in the row of the tables listing them. Both site names and resources can be filtered with the use of a multicriteria filter. An arbitrary number of conditions can be added to the filter with "and/or" and negation operators. Filters can be saved and retrieved for later use (Fig. 4).

Import / Export

Georeferenced site names can be automatically imported from comma-delimited files (CSV) and exported to comma-delimited (CSV), spreadsheet (XLS), GIS (shapefile and KML) and document (PDF) formats. Georeferencing resources can also be exported to CSV, XLS and PDF.

Usage statistics

The application provides different charts that illustrate different statistics of the georeferencing activity, such as number of georeferenced site names by georeferencer, country, type and terrestrial/aquatic nature. With respect to the georeferencing resources, their number by type is given.

Look-up tables

Shared lists of terms in the form of look-up tables (key-value tables), such as authors, organisations, keywords, version qualifiers, content types, support types, site name types and unit types, can be edited and curated with specific forms.

User management and permissions

The application provides user management functionality. A user given the administrative role can add and remove users and assign editing privileges. Users can also be given geographically-bounded permissions for editing locations, facilitated by the hierarchical structure of the site names. This allows the spatial compartmentalisation of the world into different regions which can be assigned to different georeferencers and organisations.

Institutions collaboration through federated lists

Different institutions can collaborate towards a shared georeferenced gazetteer and, thus, sum up resources and divide and distribute the overall effort. Moreover, this can be combined with the corresponding permissions to assign different geographical zones to different institutions and georeferencers, taking advantage of the better direct knowledge of parts of the territory by different actors and, potentially resulting in better georeferencing. Georeferenced site names are tagged with the name of the institution of their corresponding georeferencers. Users can choose to see only the site names georeferenced by their institution or the whole set of site names from all federated institutions.

Multi-language support

The application supports multiple languages. They can be added to the list of available languages by translating a language configuration file.

Public API

Ali-Bey also provides a public application programming interface that gives access to different functionalities. Other applications can use it and perform different queries to the system, such as returning all locations within a given geometry, returning all data concerning a given locality etc.

A working example that uses Ali-Bey's API can be found at https://www.bioexplora.cat/en/geocoding. This is a gateway to the Ali-Bey's database of georeferenced site names of the MCNB. This web page also provides the possibility for users to give comments to improve the georeferencing.

Containerised deployment

Ali-Bey can be deployed as a multi-container docker application. This allows for the easy set-up in any operating system which supports Docker, avoiding conflicts with existing libraries and language interpreter versions in the hosting operating system.

## Web location (URIs)

Homepage: https://www.bioexplora.cat/en/geocoding (API)

## Technical specification

Platform: Django 1.11.6, Bootstrap 3.3.7, jQuery 3.3.1

Programming language: Python 3.6, HTML5, JavaScript

Operational system: Linux (with Nginx 1.19.0, Gunicorn 19.9.0, Tomcat 8.5.41, GeoServer 2.19.2, PostgreSQL 10.7, PostGIS 2.5), Docker 20.10.2

Interface language: English, multilanguage support

Service endpoint: https://www.bioexplora.cat/en/geocoding (API)

## Repository

Type: GitHub. Ali-Bey is distributed as follows in three separate repositories, one for the Django application, one for the dockerised deployment of the application and one for the Ali-Bey API. 1) Ali-Bey application: Public repository of the Ali-Bey georeferencing platform, 2) Ali-Bey deployment: Public repository for the definition and deployment of the docker container for the application and 3) Ali-Bey API: Public repository for the Application Programming Interface of Ali-Bey.

Browse URI: 1) URL: https://github.com/aescobarr/mcnb-alibey, 2) https://github.com/aescobarr/mcnb-alibey-docker, 3) https://github.com/aescobarr/mcnb-alibey-api

## Usage licence

### Usage licence

Other

### IP rights notes

GNU General Public License v3.0

## Implementation

### Implements specification

The application is developed around the Model-View-Controller pattern on a three-tier architecture.

Presentation tier

Ali-Bey can be accessed with any web browser, although the most common ones are recommended (Chrome, Firefox, Safari, Edge). The user interface is implemented in HTML5 and JavaScript using the Bootstrap CSS framework (https://getbootstrap.com) and the jQuery JavaScript library (https://jquery.com). Map navigation is provided via the Leaflet JavaScript library (https://leafletjs.com). The user interface is responsive, it adapts itself to different device screen sizes. Ali-Bey uses the Nginx (https://www.nginx.com) web server as a proxy for the Django application and renders HTML pages to the user.

Application tier

The application layer is implemented in Python using the Django web framework (https://www.djangoproject.com). Django provides data access, processing and presentation functionality. Spatial operations, such as centroid calculation, are done using PostGIS (https://postgis.net) and the Geospatial Data Abstraction Library (GDAL, https://gdal.org). Cartography is served through the Web Mapping Service Interface Standard (WMS), from either locally-stored geospatial files or externally accessible WMS sites. Internal WMS functionality is provided by GeoServer (http://geoserver.org), an open source server for sharing geospatial data. Apache Tomcat (https://tomcat.apache.org) acts as an internal application server for GeoServer, the WMS map server.

Data tier

The data layer is composed of two main parts, the geodatabase and the digital cartography. The geodatabase is a PostgreSQL (https://www.postgresql.org) with the PostGIS (https://postgis.net) spatial relational database extension. Digital cartographic data, corresponding to internal georeferencing resources, are stored as geospatial data files.

API

Although Ali-Bey is meant to be an internal tool for georeferencing within an institution holding natural history collections, other applications can be built using the public API to give access to the georeferenced site names database. The *Museu de Ciències Naturals de Barcelona* provides public access to its Ali-Bey database of georeferenced site names through a web page which implements the Ali-Bey API.

The application programming interface is based on the REST (REpresentational State Transfer) architectural style which allows the use of HTTP methods to perform create, retrieve, update and delete operations. Currently, in the Ali-Bey API only retrieve operations have been implemented.

Dockerised multi-container platform

**Figure 5.**
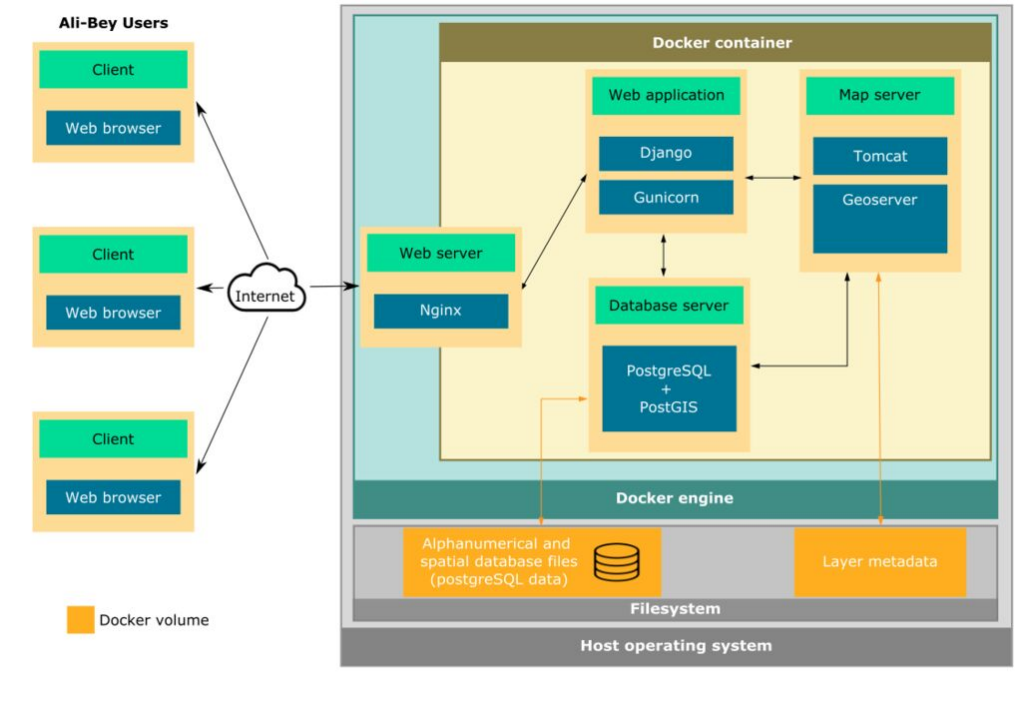
Application architecture of Ali-Bey deployed as a Docker multi-container application. A user connects with a web browser through the Internet to an Nginx web server that gives access to the application. The application is implemented with the Django web framework that interacts with the map server Geoserver and with the PostgreSQL/PostGIS database with the help of the Python HTTP server Gunicorn. The PostgreSQL alphanumeric and spatial database files and the layer metadata are all persistently stored in the host filesystem, configured as a Docker volume.

Ali-Bey uses the Docker containerisation platform (https://www.docker.com) to bundle its components and provide for an easy deployment (Fig. 5). The docker container is composed of four services bundled together through a docker-compose.yml file: database, web server, map server and web application. In order to make alphanumeric and spatial data persistent, the datafiles of the PostgreSQL database are stored in a directory of the host filesystem which is configured as a Docker volume (Figure 5). Two open ports are needed to run the application. The default ones are 8080 for the map server and 1337 for the web application. However, these can be changed to suit each deployment requirement.
